# White matter hyperintensities in autopsy-confirmed frontotemporal lobar degeneration and Alzheimer’s disease

**DOI:** 10.1186/s13195-021-00869-6

**Published:** 2021-07-13

**Authors:** Philippe Desmarais, Andrew F. Gao, Krista Lanctôt, Ekaterina Rogaeva, Joel Ramirez, Nathan Herrmann, Donald T. Stuss, Sandra E. Black, Julia Keith, Mario Masellis

**Affiliations:** 1 Cognitive & Movement Disorders Clinic, Sunnybrook Health Sciences Centre, 2075 Bayview Ave., Room A4 42, Toronto, ON M4N 3M5 Canada; 2 L.C. Campbell Cognitive Neurology Research Unit, Sunnybrook Health Sciences Centre, Toronto, ON Canada; 3 Hurvitz Brain Sciences Program, Sunnybrook Research Institute, University of Toronto, Toronto, ON Canada; 4 Division of Neurology, Department of Medicine, University of Toronto, Toronto, ON Canada; 5 Laboratory Medicine and Molecular Diagnostics, Sunnybrook Health Sciences Centre, Laboratory Medicine and Pathobiology, University of Toronto, Toronto, ON Canada; 6 Department of Psychiatry, University of Toronto, Toronto, ON Canada; 7 Tanz Centre for Research in Neurodegenerative Diseases, University of Toronto, Toronto, ON Canada; 8 Institute of Medical Science, University of Toronto, Toronto, ON Canada

**Keywords:** Alzheimer’s disease, Frontotemporal lobar degeneration, White matter hyperintensity, Magnetic resonance imaging, Neuropsychiatric symptoms, Neuropathology

## Abstract

**Background:**

We aimed to systematically describe the burden and distribution of white matter hyperintensities (WMH) and investigate correlations with neuropsychiatric symptoms in pathologically proven Alzheimer’s disease (AD) and frontotemporal lobar degeneration (FTLD).

**Methods:**

Autopsy-confirmed cases were identified from the Sunnybrook Dementia Study, including 15 cases of AD and 58 cases of FTLD (22 FTLD-TDP cases; 10 FTLD-Tau [Pick’s] cases; 11 FTLD-Tau Corticobasal Degeneration cases; and 15 FTLD-Tau Progressive Supranuclear Palsy cases). Healthy matched controls (*n* = 35) were included for comparison purposes. Data analyses included ANCOVA to compare the burden of WMH on antemortem brain MRI between groups, adjusted linear regression models to identify associations between WMH burden and neuropsychiatric symptoms, and image-guided pathology review of selected areas of WMH from each pathologic group.

**Results:**

Burden and regional distribution of WMH differed significantly between neuropathological groups (*F*_5,77_ = 2.67, *P’* = 0.029), with the FTLD-TDP group having the highest mean volume globally (8032 ± 8889 mm^3^) and in frontal regions (4897 ± 6163 mm^3^). The AD group had the highest mean volume in occipital regions (468 ± 420 mm^3^). Total score on the Neuropsychiatric Inventory correlated with bilateral frontal WMH volume (β = 0.330, *P* = 0.006), depression correlated with bilateral occipital WMH volume (β = 0.401, *P* < 0.001), and apathy correlated with bilateral frontal WMH volume (β = 0.311, *P* = 0.009), all corrected for the false discovery rate. Image-guided neuropathological assessment of selected cases with the highest burden of WMH in each pathologic group revealed presence of severe gliosis, myelin pallor, and axonal loss, but with no distinguishing features indicative of the underlying proteinopathy.

**Conclusions:**

These findings suggest that WMH are associated with neuropsychiatric manifestations in AD and FTLD and that WMH burden and regional distribution in neurodegenerative disorders differ according to the underlying neuropathological processes.

**Supplementary Information:**

The online version contains supplementary material available at 10.1186/s13195-021-00869-6.

## Background

Regional gray matter atrophy has been linked to the clinical expression of Alzheimer’s disease (AD) and frontotemporal lobar degeneration (FTLD) [1–3]. Antemortem hippocampal atrophy has been associated with episodic memory deficits [4] while orbitofrontal atrophy has been linked to disinhibition [5]. While regional gray matter atrophy represents an important biomarker for these disorders, these changes occur late in their respective pathological cascades and therefore development of other imaging biomarkers is warranted.

Changes in white matter, such as hyperintensities on T2-weighted MRI sequences, have gained interest as potential biomarkers in neurodegenerative disorders. Historically, white matter hyperintensities (WMH) have been associated with cerebrovascular disease, vascular risk factors, and age, and to parallel global and regional cortical atrophy [6]. More recently, WMH in AD and congophilic amyloid angiopathy (CAA) may represent microvascular dysfunction secondary to amyloid β deposition in the cerebral vasculature [7] as well as venous collagenosis [8], independent of age and vascular risk factors. Furthermore, WMH may be associated with clinical manifestations. In prospective longitudinal studies of older cognitively normal adults and AD patients, periventricular WMH correlated negatively with mental processing speed, while left temporal WMH correlated negatively with memory performance [9, 10]. However, neural correlates of WMH have not been extensively and rigorously studied in pathologically proven cases of AD and FTLD.

Mapping the distribution and burden of WMH in AD and FTLD may further our understanding of the underlying pathological mechanisms of these disorders. In this study, we aimed to: (1) using antemortem MRI, describe WMH burden and distribution in these neuropathological entities, (2) investigate brain WMH-behavioural correlates of neuropsychiatric manifestations, and (3) perform an image-guided, detailed neuropathological assessment of regions where WMH were observed in representative cases from each of the neuropathological subtypes with the highest burden of WMH.

## Methods

### Participants

This research was carried out as part of the Sunnybrook Dementia Study, a prospective longitudinal cohort study of cognitively normal ageing, mild cognitive impairment, and neurodegenerative dementias conducted at Sunnybrook Health Sciences Centre, University of Toronto (ClinicalTrials.gov: NCT01800214). The design and methods have been previously published [11]. The study was approved by the Research Ethics Board at Sunnybrook Health Sciences Centre and all participants, or their caregivers when appropriate, provided written informed consent, in accordance with the Declaration of Helsinki.

Consecutive deceased participants with autopsy-confirmation of pure FTLD (i.e., without other pathological comorbidity) were retrospectively identified, including cases of FTLD due to Pick’s disease (i.e., FTLD-Tau [Pick’s]), TDP-43 proteinopathy (i.e., FTLD-TDP), Progressive Supranuclear Palsy (i.e., FTLD-Tau [PSP]), and Corticobasal Degeneration (i.e., FTLD-Tau [CBD]). Cognitively impaired participants with autopsy-confirmation of pure Alzheimer’s pathology without other proteinopathies (e.g., synucleinopathy or FTLD-TDP) or large vessel cerebrovascular disease were consecutively selected. Healthy matched controls were randomly selected from the Sunnybrook Dementia Study cohort and included in the present study for comparison purposes for the antemortem MRI study. For enrolment in the study, these participants had to be between the age of 40 and 90, be fluent in English, completed 8 years of education or higher, and have no significant memory complaints. They were excluded if they were being treated or had a history of being treated for a psychiatric or neurological illness (i.e., other than primary neurodegenerative diagnoses), history of alcohol or substance abuse or dependence, were currently using psychoactive medications not indicated for their primary neurodegenerative diagnoses, or had medical contraindications to MRI.

### Genetic studies

Genomic DNA was extracted from whole blood using Qiagen kits. DNA from participants with a clinical diagnosis of a frontotemporal dementia spectrum disorder was screened for pathogenic mutations known to cause FTLD: *C9orf72* [12], *GRN* [13], and *MAPT* [14]. A pathogenic expansion of *C9orf72* was considered as having more than 30 repeats. All selected cases of AD were sporadic in nature, free of mutations in the *APP*, *PSEN1*, and *PSEN2* genes [14].

### Neuropathology

Autopsies limited to the brain and spinal cord were performed by an experienced neuropathologist (author JK). Neuropathological diagnoses and staging were made for the primary disease process and any co-existing neurodegenerative phenomena at the time of original autopsy based on a standardized blocking and staining protocol for dementia, applying consensus criteria for AD [15–18] and FTLD [19, 20]. Cases were included in the study and assigned into the following neuropathological diagnostic categories based on the original autopsy reports: (i) AD (*n* = 15), (ii) FTLD-Tau (Pick’s) (*n* = 10), (iii) FTLD-Tau (PSP) (*n* = 15), (iv) FTLD-Tau (CBD) (*n* = 11), and (v) FTLD-TDP (*n* = 22).

The pathologic classification of FTLD-TDP has evolved in recent years; thus, these cases were subjected to a central pathology review. Slides from the original autopsy were retrieved from the Sunnybrook pathology archive and reviewed by authors JK and AG. Additional slides were cut from selected original tissue blocks and stained with antibodies for TDP-43, alpha-synuclein, tau (AT8), and/or p62 (antibody information provided in the Additional file 1). Based on this central pathology review, cases within the FTLD-TDP category were re-classified using the harmonized consensus criteria for FTLD-TDP pathology as types A-D [21, 22].

Cases were excluded from the study if (i) neuropathological diagnosis could not be accurately assessed (*n* = 1), (ii) immunohistochemical staining examination was incomplete and could not be retrospectively completed (*n* = 2), and (iii) multiple co-morbid neuropathologies were present, none of which could be assigned unequivocally as the predominant cause of dementia (*n* = 25; 10 cases of FTLD had concomitant alpha-synucleinopathy, amyloid plaques, or infarcts, and 15 cases of AD had concomitant alpha-synucleinopathy, infarcts, or TDP43).

### MRI acquisition and analysis

All participants underwent MRI on a 1.5 Tesla GE Signa (Milwaukee, WI, USA) system in compliance with consensus panel imaging recommendations for studies examining vascular cognitive impairment [23]. The following sequences were used for volumetric analysis: T1-weighted-axial three-dimensional (3D) Spoiled Gradient Recalled Echo (SPGR): 5 ms echo time (TE), 35 ms repetition time (TR), 1 number of excitations (NEX), 35^o^ flip angle, 22 × 16.5cm (FOV), 0.859 × 0.859 mm in-plane resolution, 1.2 to 1.4 mm slice thickness depending on head-size, and a whole head interleaved proton density and T2 (interleaved axial dual-echo spin echo: TEs of 30 and 80 ms, 3 s TR, 0.5 NEX, 20 × 20 cm FOV, 0.781 × 0.781 mm in-plane resolution, 3-mm slice thickness with no gaps between slices).

MR images were analyzed with the semi-automatic brain region extraction (SABRE) and Lesion Explorer (LE) processing pipeline [24], which permits semi-automatized segmentation and parcellation procedures and to obtain regionalized and whole-brain volumetrics for normal appearing tissues and WMH. An automated 3D connectivity algorithm was applied to segment periventricular from deep WMH. Volumes for gray matter, normal appearing white matter, and WMH were obtained in 26 regions of interest, 13 per hemisphere (frontal: superior, middle, inferior, medial inferior, medial superior, medial middle; parietal: superior, inferior; occipital; temporal: anterior, posterior; basal ganglia/thalamus: anterior, posterior). Intracranial volumetric data (gray matter and WMH) were normalized for total intracranial volume (TIV). For analysis, all WMH values were log-transformed after normalizing for TIV due to their known skewed distribution [24].

### Neuropsychological and neuropsychiatric assessments

Participants underwent a standardized clinical evaluation at baseline within 12 weeks of MRI acquisition. This comprised a medical history, physical examination, and a neuropsychological and neuropsychiatric battery [25]. The following vascular risk factors were collected: hypertension, hyperlipidemia, diabetes mellitus, and history of stroke and/or transient ischemic attack. For the purpose of this study, cognitive and neuropsychiatric testing results were retrieved for the following: (i) the Mini-Mental Status Examination (MMSE) [26], (ii) the Dementia Rating Scale (DRS) [27], and (iii) the Neuropsychiatric Inventory (NPI) [28]. For the latter, the total score (maximum of 144 points) and the 12 items, i.e., neuropsychiatric symptom subscores (maximum of 12 points for each item), were obtained, as well as the caregiver distress subscore (maximum of 60 points).

### Image-guided neuropathology review of white matter regions with highest burden of WMH

For each neuropathologic group (FTLD-TDP, FTLD-Tau [PSP], FTLD-Tau [CBD], and FTLD-Tau [Pick’s], AD with CAA, and AD without CAA), antemortem T2-weighted MRI images were examined to determine the one case per group with the greatest volume of WMH. These cases subsequently underwent further pathologic evaluation of the affected white matter using annotated coronal MRI images as a guide (see Additional Figure 1, in Additional file 1). One periventricular white matter region that was heavily affected by WMH on MRI was identified within the previous coronally dissected formalin-fixed archived cadaveric brain tissue. For one case, no white matter region of interest could be found in the remaining brain tissue. For the other seven cases, this single region of interest was sampled. FFPE sections were cut at 6 microns and stained with H&E/LFB as well as immunohistochemistry for neurofilament, GFAP, CD68, Tau (AT8), and TDP43 (antibody information provided in Additional file 1). These slides were scanned at 40X on an Aperio ScanScope AT Digital Pathology Slide Scanner and examined digitally synchronously by two experienced neuropathologists (authors AG and JK) who were blinded to the neuropathologic diagnosis/group of each case. Correlating the H&E/LFB stained slide to the annotated coronal MRI image, the white matter region of interest was located within the sampled tissue. On H&E/LFB, pallor of myelin staining was assessed semi-quantitatively (0–3: none, mild, moderate, severe) using subcortical U-fibers as the internal control for none. Arteriolosclerosis, hemosiderin deposition, and collagenosis of the small and large caliber periventricular veins were determined to be present or absent. TDP43 and tau (AT8)-positive inclusions, axonal loss (neurofilament), gliosis (GFAP), and macrophage/microglial infiltration/activation (CD68) were assessed semi-quantitatively (0–3) on immunohistochemistry.

### Statistical analyses

We compared baseline characteristics between each of the neuropathological groups and the healthy control group using ANOVA with post hoc Bonferroni tests for continuous, normally distributed variables, *χ*^2^/Fisher exact tests for categorical/dichotomous variables, respectively, and Kruskal-Wallis with post hoc Mann-Whitney U tests for non-normally distributed data. Differences in total and regional volumes of WMH on T2-weighted imaging (i.e., dependent variables) among neuropathological groups and the healthy control group were assessed by using ANCOVA, controlling for age at imaging and vascular risk factors.

We also assessed for the association between regional WMH volumes and corresponding regional gray matter volumes using multiple linear regression analyses, controlling for age, education, sex, and vascular risk factors, with regional WMH volume as the independent variable and the corresponding grey matter volume as the dependent variable.

We conducted linear regressions to assess for associations between global and regional WMH volumes and scores on the NPI (total scores and 12 subscale scores) across all neuropathological groups. For the linear regressions, a model was designed a priori and contained age, sex, vascular risk factors, and corresponding regional gray matter volumes as covariates, with WMH volume as the independent variable and NPI score as the dependent variable. Considering (1) the exploratory nature of our study, (2) that NPI subscores are highly correlated with each other, and (3) that regional WMH volumes are also highly correlated with each other, the Benjamini-Hochberg procedure was used to control for the false discovery rate across all regression analyses for each brain region, with a false discovery rate (FDR; *Q*) set at 0.10. A Bonferroni correction is too strict due to the non-independence of these variables as outlined above. Statistical analyses were performed with the Statistical Package for the Social Sciences, version 24.0.

## Results

### Participant characteristics

A summary of demographic and clinical characteristics of participants with the various neuropathological diagnoses is provided in Table 1 and in the Additional Table 1 (Additional file 1). No significant differences were present except for, as expected, overall performance on the cognitive tests, specifically on the MMSE and on the DRS. These were significantly lower in the pathologic neurodegenerative disease groups compared to the healthy control group (*F*_5,95_ = 10.27, *P* < 0.0001, and *F*_5,90_ = 13.42, *P* < 0.0001, respectively), but did not differ significantly between pathologic neurodegenerative disease groups (*F*_4,61_ = 1.69, *P* = 0.112 and *F*_4,56_ = 0.59, *P* = 0.680 for the mean scores on the MMSE and DRS, respectively). There were six cases of FTLD with pathogenic mutations: 4 *GRN* and 2 *C9orf72* mutation carriers.

**Table 1:** Demographic and clinical characteristics

Characteristics	FTLD-TDP (***n*** = 22)	FTLD-tau (Pick's) (***n*** = 10)	FTLD-tau (CBD) (***n*** = 11)	FTLD-tau (PSP) (***n*** = 15)	AD (***n*** = 15)	HC (***n*** = 35)	***P***-values
Age at baseline	66.5 (8.7)	67.4 (9.6)	67.4 (6.6)	71.4 (5.3)	69.1 (10.0)	71.2 (7.8)	0.278
Age at onset of symptoms	63.2 (8.5)	63.5 (11.1)	63.9 (6.0)	68.3 (5.8)	65.6 (10.5)	..	0.454
Disease duration at baseline assessment (years)	3.9 (3.7)	3.9 (3.7)	3.6 (1.7)	3.1 (1.7)	3.6 (2.2)	..	0.903
Age at imaging	68.2 (7.6)	67.8 (9.8)	68.1 (6.2)	72.0 (5.0)	69.5 (10.5)	71.6 (7.5)	0.059
Age at death	73.1 (10.5)	74.9 (8.0)	71.1 (5.9)	76.4 (5.3)	74.5 (10.5)	..	0.612
Female	10 (45%)	4 (40%)	8 (73%)	6 (40%)	5 (33%)	14 (40%)	0.455
Handedness (R/L/A)	17/4/1	7/2/1	10/1/0	13/1/1	15/0/0	16/0/1	0.512
Education (years)	15.0 (3.2)	15.4 (4.1)	13.5 (3.2)	15.3 (3.2)	16.0 (5.5)	14.4 (3.3)	0.570
MMSE (/30)	22.7 (6.3)	16.9 (10.6)	21.9 (5.8)	24.1 (6.2)	19.9 (6.4)	28.9 (0.9)	<0.0001*
DRS (/144)	108.0 (21.1)	102.6 (36.9)	103.4 (25.2)	115.4 (18.9)	110.9 (21.2)	140.5 (2.6)	<0.0001*
Total NPI (/144)	25.1 (10.9)	42.2 (23.7)	18.3 (18.6)	14.7 (15.1)	11.9 (8.0)	..	<0.0001*
Vascular risk factors							
Hypertension	6 (27%)	2 (20%)	3 (27%)	5 (33%)	4 (27%)	9 (26%)	0.972
Hyperlipidemia	5 (23%)	0 (0%)	3 (27%)	2 (13%)	6 (40%)	1 (3%)	0.168
Diabetes mellitus	0 (0%)	1 (10%)	2 (18%)	0 (0%)	0 (0%)	0 (0%)	0.177
History of stroke/TIA	2 (10%)	1 (10%)	0 (0%)	0 (0%)	1 (6.6%)	0 (0%)	0.705
Total WMH (mm^3^)	8032 (8889)	3088 (3238)	4179 (4664)	4695 (4872)	4840 (5045)	3053 (11947)	0.058

Data are n (%) and mean (± standard deviation). All percentages were rounded to the nearest whole number

Brain imaging acquisition was performed within 12 weeks of participant’s baseline assessment

*Significant difference between groups (*P* < 0.05) on ANOVA with post hoc Bonferroni tests for continuous variables, *χ*^2^/Fisher Exact Tests for categorical/dichotomous variables, respectively, and Kruskal-Wallis with post hoc Mann-Whitney U tests for non-normally distributed data

*Abbreviations: AD* Alzheimer’s disease, *CBD* corticobasal degeneration, *DRS* Dementia Rating Scale, *FTLD* frontotemporal lobar degeneration, *HC* healthy controls, *MMSE* Mini-Mental State Examination, *NPI* Neuropsychiatric Inventory, *PSP* progressive supranuclear palsy, *TIA* transient ischemic attack, *WMH* white matter hyperintensity

### Neuropsychiatric symptoms

**Fig. 1:**
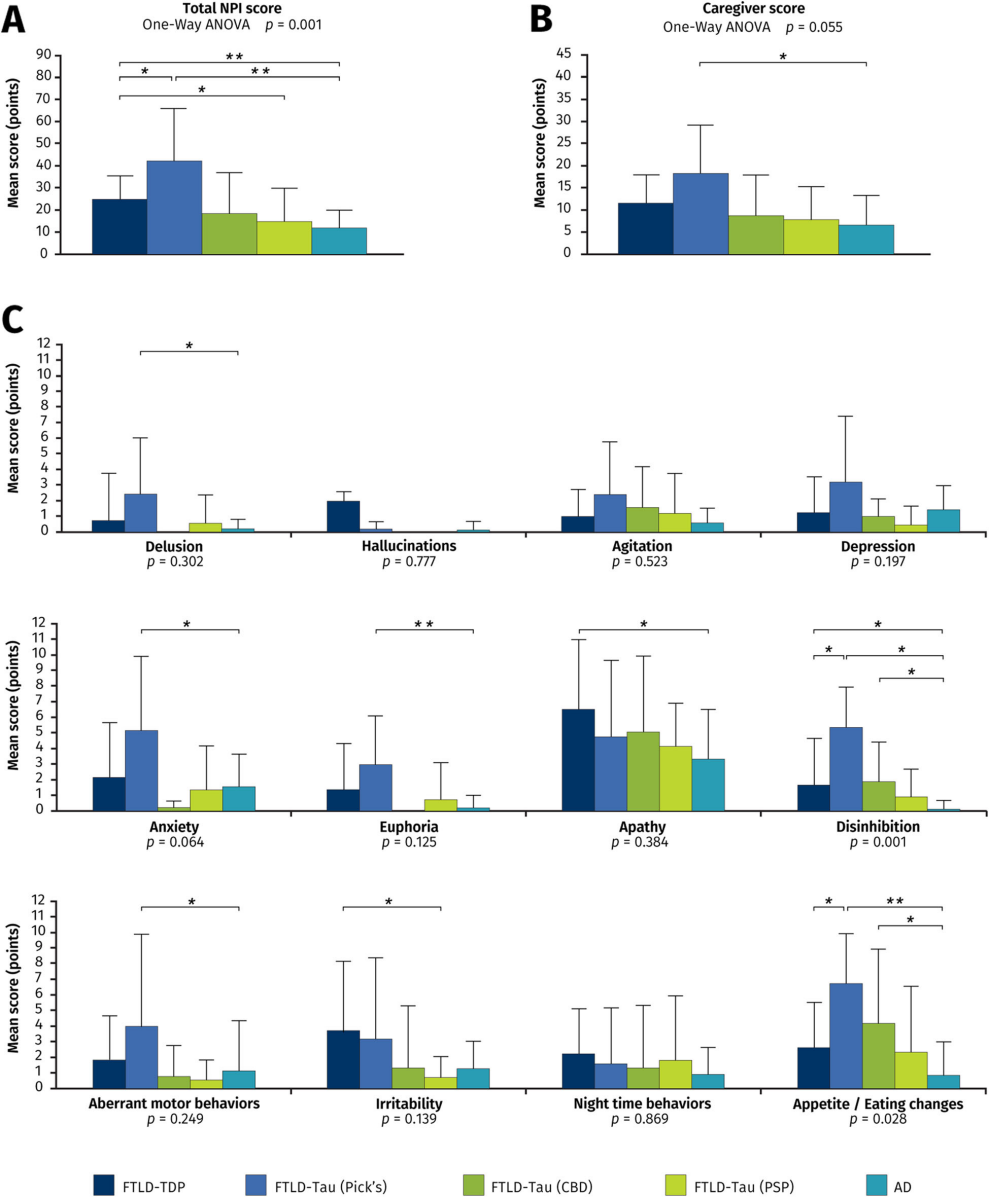
Neuropsychiatric symptoms at baseline. Mean score and standard deviation on the Neuropsychiatric Inventory (NPI) for all of the pathological subgroups. **A** Total NPI score. **B** Caregiver score. **C** Subscale items. *P* values for differences between subgroups (ANOVA) are found underneath graphs. All bars in the figure are significant differences between each pair with * = *P* < 0.05 and ** = *P* < 0.005. *Abbreviations: AD* Alzheimer’s disease, *CBD* corticobasal degeneration, *FTLD* frontotemporal lobar degeneration, *PSP* progressive supranuclear palsy

Baseline neuropsychiatric symptom profile of participants according to neuropathological diagnosis is shown in Fig. 1. Overall score on the NPI differed between groups (*F*_4,45_ = 4.39, *P* < 0.0001), with FTLD-Tau (Pick’s) (42.2 ± 23.7 points) and FTLD-TDP (25.1 ± 10.9 points) having significantly higher mean scores than for the AD group (11.9 ± 8.0 points, *P* < 0.001 for both comparisons). Apathy was the most prevalent neuropsychiatric manifestation in all neuropathological groups, while hallucinations and delusions were infrequently reported symptoms. Of all the subgroups, FTLD-Tau due to Pick’s disease had the highest caregiver burden mean score (18.2 ± 10.8 points) as well as the highest mean scores for 8 of the 12 NPI subscales.

### WMH volumetrics among neuropathological groups and controls

**Fig. 2:**
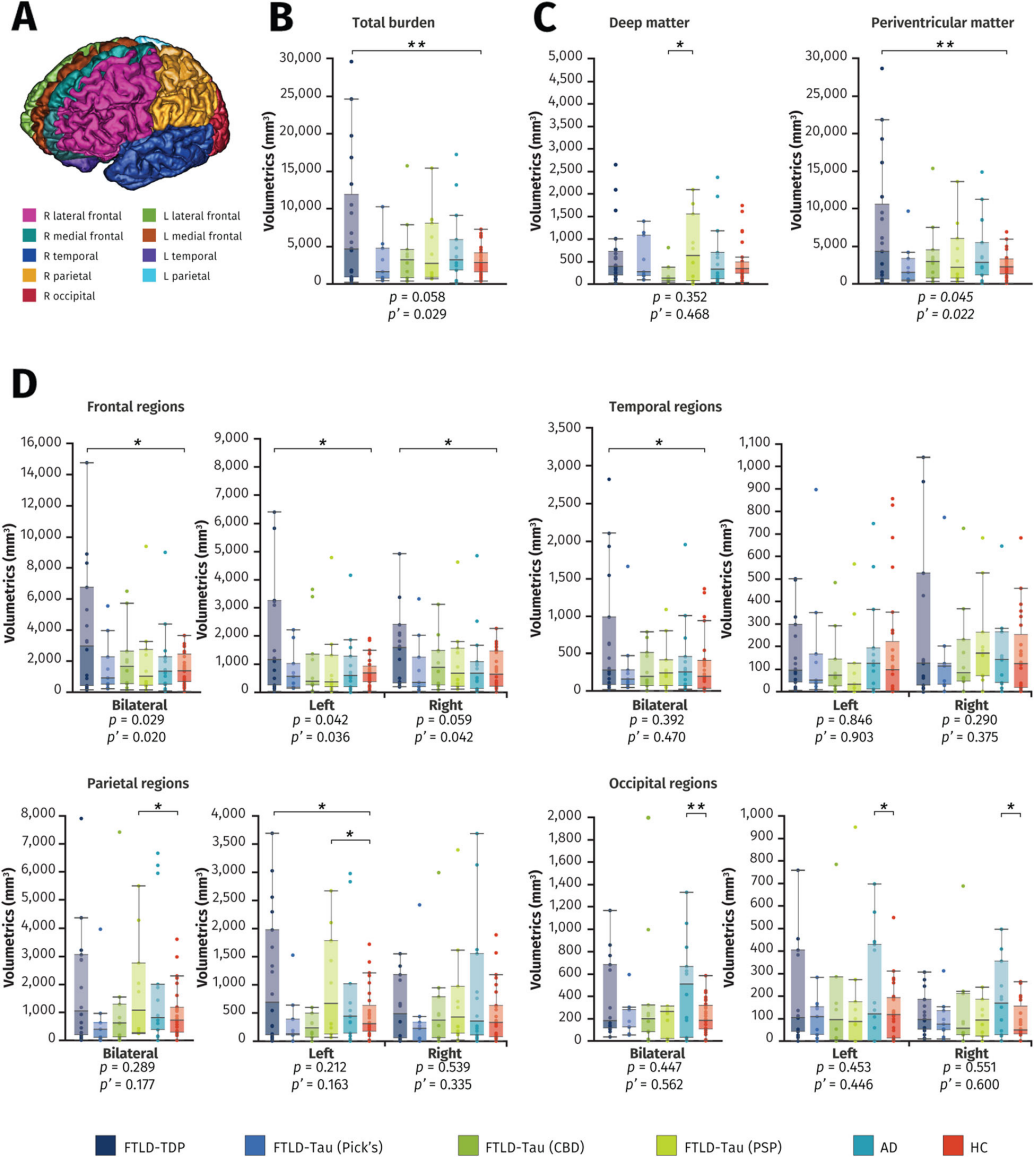
Comparison of white matter hyperintensity volumes between groups. Box plots show white matter hyperintensity volumes on T2-weighted images according to neuropathological diagnosis, with lower and upper hinges of each boxplot corresponding to 25th and 75th percentiles of data. **A** SABRE parcellation of brain regions. **B** Total intracranial white matter hyperintensity burden. **C** Deep white matter and periventricular white matter hyperintensity burden. **D** Regional white matter hyperintensity volumetrics. Underneath the graphs are *P* values for differences between subgroups (ANOVA) and *P*’ values for differences between subgroups adjusting for age and vascular risk factors (ANCOVA). All bars in the figure are significant differences between each pair with * = *P* < 0.05 and ** = *P* < 0.005, corrected for age at imaging and vascular risk factors. *Abbreviations: AD* Alzheimer’s disease, *CBD* corticobasal degeneration, *FTLD* frontotemporal lobar degeneration, *HC* healthy controls, *PSP* progressive supranuclear palsy

In the healthy control group, total WMH and periventricular WMH volumes were significantly associated with age (*r* = 0.481, *P* = 0.009 and *r* = 0.490, *P* = 0.008, respectively) but not for deep WMH (*r* = 0.119, *P* = 0.546). After adjusting for vascular risk factors and age at time of imaging, WMH burden and cerebral distribution significantly differed between neuropathological groups and the healthy control group (Fig. 2). There were significant differences between groups in the total burden of WMH (*F*_5,77_ = 2.67, *P’* = 0.029), with FTLD-TDP having the highest mean volume (8032 ± 8889 mm^3^) and FTLD-Tau (Pick’s) having the lowest mean volume (3088. ± 3238 mm^3^). Groups also differed significantly in volumes of WMH in the periventricular region (*F*_5,77_ = 2.82, *P’* = 0.022) and frontal regions (*F*_5,77_ = 2.59, *P’* = 0.020), more specifically in lateral frontal regions (*F*_5,77_ = 2.13, *P’* = 0.029), with FTLD-TDP having the highest burden in these regions (7404 ± 8539 mm^3^; 4897 ± 6163 mm^3^; and 3761 ± 5069 mm^3^, respectively). The AD group had the highest mean volume of WMH in the occipital region (468 ± 420 mm^3^; Fig. 2). Post hoc analyses revealed that AD with CAA was associated with significantly higher burden of WMH in the bilateral parietal region than for AD without CAA (3241 ± 2911 mm^3^ vs. 672 ± 664 mm^3^, *P* = 0.042). For FTLD-TDP, Harmonized type was determined for 19 of 22 cases: type A (*n* = 11), type B (*n* = 2), mixed type A+B (*n* = 2), and type C (*n* = 4). We did not have adequate power to run statistical analyses due to small FTLD-TDP subgroup sizes.

Multiple linear regressions between regional WMH volumes and corresponding gray matter volumes for each pathologic neurodegenerative disease group and healthy controls were performed. There were no significant associations found in the healthy control and AD groups. In FTLD, increased regional volumes of WMH were associated with decreased volumes of gray matter in the corresponding area in the right frontal region (β = −0.425, *P* = 0.004), in the right lateral frontal region (β = −0.345, *P* = 0.034), in the right parietal region (β = −0.299, *P* = 0.049), and in the right occipital region (β = −0.462, *P* = 0.002). All of these correlations survived FDR correction.

### Neuropsychiatric symptoms and WMH volumetrics

Across the entire pathologically confirmed neurodegenerative disease cohort, positive associations between neuropsychiatric symptoms and total and regional burden of WMH (Tables 2, 3, and 4) were observed; specifically, between the total NPI score and WMH volume in frontal regions (bilateral frontal region, β = 0.330, *P* = 0.006), with the strongest correlation found in the right lateral frontal region (β = 0.339, *P* = 0.008). Regarding specific neuropsychiatric manifestations, significant positive correlations were identified for the depression, apathy, euphoria, and night-time behaviors NPI subscores. For depression, increased bilateral occipital and right parietal WMH were associated with higher mean scores (β = 0.401, *P* < 0.001; and β = 0.326, *P* = 0.007, respectively). For euphoria, a significant correlation was identified with WMH in deep white matter (β = 0.317, *P* = 0.006). For apathy, several significant positive correlations were identified with WMH burden in frontal regions, with the strongest correlation being in the bilateral frontal region (β = 0.311, *P* = 0.009). Similarly, several significant positive correlations were identified with night-time behaviors and WMH in frontal regions, with the strongest correlation being in the right lateral frontal region (β = 0.390, *P* = 0.003). These regression analyses were corrected for age, sex, vascular risk factors, and corresponding regional gray matter volumes.

**Table 2:** Linear regression between white matter hyperintensities and neuropsychiatric symptoms across all subgroups

Neuropsychiatric symptom	Total NPI			Delusion			Hallucination			Agitation			Depression		
Region	β coefficient	Unadjusted *P* value	FDR q value	β coefficient	Unadjusted *P* value	FDR q value	β coefficient	Unadjusted *P* value	FDR q value	β coefficient	Unadjusted *P* value	FDR q value	β coefficient	Unadjusted *P* value	FDR q value
**Total**	0.212	0.080	0.023	0.138	0.271	0.038	−0.116	0.358	0.054	0.077	0.540	0.085	0.267	0.028	0.008
**Deep matter**	0.197	0.093	0.023	0.161	0.186	0.038	−0.083	0.495	0.077	−0.040	0.741	0.100	0.115	0.337	0.062
**Periventricular**	0.201	0.099	0.023	0.126	0.319	0.038	−0.113	0.372	0.054	0.090	0.478	0.062	0.273	0.026	0.008
**Frontal**	0.330*	0.006*	0.015	0.207	0.099	0.054	0.056	0.956	0.100	0.121	0.338	0.085	0.147	0.236	0.069
Left	0.267	0.034	0.023	0.257	0.039	0.031	−0.022	0.862	0.100	0.102	0.410	0.085	0.129	0.300	0.069
Right	0.282	0.017	0.015	0.148	0.253	0.062	0.042	0.741	0.092	0.106	0.409	0.085	0.138	0.279	0.069
**Frontal—medial**	0.089	0.493	0.046	−0.085	0.531	0.054	0.135	0.314	0.023	0.019	0.892	0.092	−0.054	0.688	0.069
Left	0.052	0.688	0.046	−0.014	0.917	0.085	0.124	0.357	0.031	0.018	0.896	0.077	0.026	0.851	0.069
Right	0.095	0.414	0.038	−0.057	0.661	0.085	0.093	0.474	0.046	0.059	0.654	0.077	−0.145	0.272	0.031
**Frontal—lateral**	0.314*	0.012*	0.008	0.251	0.056	0.031	−0.075	0.566	0.092	0.098	0.470	0.085	0.148	0.258	0.077
Left	0.263	0.042	0.023	0.280	0.034	0.015	−0.087	0.508	0.085	0.065	0.630	0.100	0.087	0.519	0.092
Right	0.339*	0.008*	0.015	0.225	0.097	0.046	−0.027	0.839	0.100	0.097	0.484	0.085	0.250	0.067	0.031
**Temporal**	0.078	0.509	0.069	0.090	0.462	0.062	−0.097	0.426	0.046	0.034	0.782	0.085	0.166	0.166	0.008
Left	−0.108	0.218	0.008	−0.055	0.651	0.062	−0.032	0.787	0.077	−0.086	0.481	0.023	−0.013	0.915	0.092
Right	0.195	0.103	0.031	0.139	0.248	0.054	−0.094	0.439	0.077	0.095	0.428	0.069	0.240	0.040	0.008
**Parietal**	0.109	0.364	0.023	0.039	0.752	0.085	−0.080	0.519	0.038	0.078	0.529	0.046	0.252	0.036	0.008
Left	0.097	0.423	0.046	0.053	0.663	0.062	−0.070	0.567	0.054	0.125	0.303	0.015	0.120	0.319	0.023
Right	0.125	0.308	0.031	−0.004	0.974	0.100	−0.058	0.643	0.062	0.022	0.861	0.069	0.326*	0.007*	0.008
**Occipital**	0.021	0.857	0.085	0.042	0.728	0.069	−0.060	0.622	0.054	0.004	0.977	0.100	0.401*	<0.001*	0.008
Left	0.039	0.747	0.077	0.053	0.660	0.054	−0.044	0.717	0.069	0.016	0.895	0.100	0.349*	0.003*	0.008
Right	0.060	0.626	0.038	0.036	0.767	0.077	−0.015	0.898	0.077	0.032	0.793	0.085	0.386*	0.001*	0.008

Associations symptoms were assessed using linear regression analyses. Estimates are presented as standardized β values to allow for comparison of effect sizes. Model: age, sex, vascular risk factors, and corresponding regional gray matter atrophy as covariates

*Significant result following Benjamini-Hochberg FDR correction for all correlational tests

*Abbreviations: FDR* false discovery rate, *NPI* Neuropsychiatric Inventory

**Table 3:** Linear regression between white matter hyperintensities and neuropsychiatric symptoms across all subgroups

Neuropsychiatric symptom	Anxiety			Euphoria			Apathy			Disinhibition		
Region	β coefficient	Unadjusted *P-*value	FDR *q*-value	β coefficient	Unadjusted *P-*value	FDR *q*-value	β coefficient	Unadjusted *P-*value	FDR *q*-value	β coefficient	Unadjusted *P-*value	FDR *q*-value
**Total**	0.056	0.653	0.100	0.109	0.373	0.062	0.256	0.033	0.015	0.105	0.401	0.069
**Deep matter**	0.119	0.321	0.054	0.317*	0.006*	0.008	0.088	0.453	0.069	0.189	0.118	0.031
**Periventricular**	0.043	0.731	0.100	0.071	0.582	0.085	0.265	0.028	0.015	0.086	0.494	0.069
**Frontal**	0.142	0.254	0.077	0.244	0.045	0.031	0.311*	0.009*	0.008	0.181	0.150	0.062
Left	0.117	0.346	0.077	0.236	0.051	0.038	0.299	0.013	0.008	0.136	0.276	0.062
Right	0.129	0.303	0.077	0.191	0.120	0.038	0.234	0.051	0.023	0.173	0.174	0.054
**Frontal – medial**	−0.008	0.951	0.100	0.232	0.081	0.008	0.041	0.752	0.085	0.059	0.669	0.062
Left	−0.153	0.253	0.023	0.287	0.027	0.008	0.077	0.561	0.038	0.029	0.829	0.062
Right	0.082	0.519	0.069	0.177	0.158	0.008	−0.011	0.929	0.100	0.085	0.510	0.062
**Frontal – lateral**	0.176	0.176	0.062	0.208	0.109	0.046	0.308*	0.013*	0.015	0.161	0.230	0.069
Left	0.193	0.146	0.046	0.217	0.099	0.031	0.279	0.032	0.008	0.119	0.377	0.069
Right	0.172	0.200	0.069	0.192	0.153	0.054	0.315*	0.013*	0.023	0.195	0.158	0.062
**Temporal**	0.095	0.426	0.054	−0.155	0.191	0.015	0.129	0.273	0.023	−0.030	0.806	0.092
Left	−0.084	0.483	0.031	−0.075	0.516	0.046	−0.047	0.695	0.069	−0.079	0.511	0.038
Right	0.184	0.122	0.046	−0.135	0.253	0.062	0.229	0.053	0.015	0.027	0.825	0.100
**Parietal**	−0.050	0.679	0.062	0.048	0.695	0.069	0.189	0.112	0.015	0.046	0.711	0.077
Left	−0.038	0.728	0.085	0.044	0.713	0.069	0.170	0.161	0.008	0.114	0.351	0.031
Right	−0.070	0.563	0.054	0.097	0.433	0.046	0.191	0.111	0.015	0.009	0.942	0.092
**Occipital**	−0.050	0.675	0.062	−0.094	0.425	0.031	0.065	0.581	0.046	−0.024	0.843	0.077
Left	−0.025	0.835	0.092	−0.110	0.358	0.015	0.069	0.571	0.038	0.078	0.524	0.031
Right	−0.045	0.709	0.069	−0.056	0.643	0.054	0.112	0.358	0.031	−0.051	0.680	0.062

Associations between regional white matter hyperintensity volumes and neuropsychiatric symptoms were assessed using linear regression analyses. Estimates are presented as standardized β values to allow for comparison of effect sizes. Model: age, sex, vascular risk factors, and corresponding regional grey matter atrophy as covariates

*Significant result following Benjamini-Hochberg FDR correction for all correlational tests

*Abbreviations: FDR* false discovery rate, *NPI* Neuropsychiatric Inventory

**Table 4:** Linear regression between white matter hyperintensities and neuropsychiatric symptoms across all subgroups

Neuropsychiatric symptom	Aberrant motor			Irritability			Night-time behaviors			Appetite/eating habit changes		
Region	β coefficient	Unadjusted *P* value	FDR q value	β coefficient	Unadjusted *P* value	FDR q value	β coefficient	Unadjusted *P* value	FDR q value	β coefficient	Unadjusted *P* value	FDR q value
**Total**	0.136	0.277	0.046	0.062	0.593	0.092	0.149	0.239	0.031	0.075	0.539	0.077
**Deep matter**	0.070	0.566	0.092	0.136	0.223	0.046	0.306*	0.011*	0.015	0.071	0.546	0.085
**Periventricular**	0.138	0.275	0.031	0.047	0.687	0.092	0.116	0.363	0.046	0.070	0.564	0.077
**Frontal**	0.241	0.052	0.046	−0.013	0.913	0.092	0.322*	0.010*	0.023	0.237	0.049	0.038
Left	0.194	0.117	0.054	−0.085	0.471	0.092	0.267	0.029	0.015	0.198	0.093	0.046
Right	0.241	0.055	0.031	0.020	0.869	0.100	0.317	0.012	0.008	0.170	0.134	0.046
**Frontal—medial**	0.114	0.403	0.031	0.044	0.729	0.077	0.154	0.261	0.015	0.096	0.464	0.038
Left	−0.051	0.708	0.054	−0.003	0.979	0.092	0.215	0.113	0.015	−0.002	0.990	0.100
Right	0.178	0.168	0.015	0.016	0.893	0.092	0.092	0.483	0.054	0.150	0.188	0.023
**Frontal—lateral**	0.225	0.087	0.038	−0.060	0.628	0.100	0.313*	0.017*	0.023	0.196	0.122	0.054
Left	0.202	0.127	0.038	−0.101	0.428	0.077	0.186	0.154	0.054	0.169	0.186	0.062
Right	0.228	0.093	0.038	0.035	0.790	0.092	0.390*	0.003*	0.015	0.141	0.253	0.077
**Temporal**	0.128	0.292	0.031	0.110	0.327	0.038	−0.014	0.907	0.100	−0.034	0.770	0.077
Left	−0.011	0.925	0.100	−0.028	0.802	0.085	−0.066	0.585	0.054	−0.100	0.404	0.015
Right	0.192	0.112	0.038	0.193	0.087	0.023	0.036	0.770	0.085	0.032	0.789	0.092
**Parietal**	0.026	0.833	0.092	0.079	0.487	0.031	0.068	0.586	0.054	−0.023	0.846	0.100
Left	0.099	0.419	0.038	0.003	0.982	0.100	0.043	0.722	0.077	−0.029	0.810	0.092
Right	−0.020	0.873	0.077	0.138	0.226	0.023	0.120	0.341	0.038	0.012	0.921	0.085
**Occipital**	−0.022	0.857	0.092	0.146	0.189	0.015	−0.094	0.442	0.038	−0.139	0.232	0.023
Left	0.063	0.602	0.046	0.034	0.769	0.085	−0.050	0.683	0.062	−0.095	0.425	0.023
Right	−0.018	0.882	0.092	0.197	0.079	0.015	−0.058	0.635	0.046	−0.111	0.353	0.023

Associations between regional white matter hyperintensity volumes and neuropsychiatric symptoms were assessed using linear regression analyses. Estimates are presented as standardized β values to allow for comparison of effect sizes. Model: age, sex, vascular risk factors, and corresponding regional grey matter atrophy as covariates

*Significant result following Benjamini-Hochberg FDR correction for all correlational tests

*Abbreviations: FDR* false discovery rate, *NPI* Neuropsychiatric Inventory

### Neuropathological findings within sampled WMH guided by MRI

**Fig. 3:**
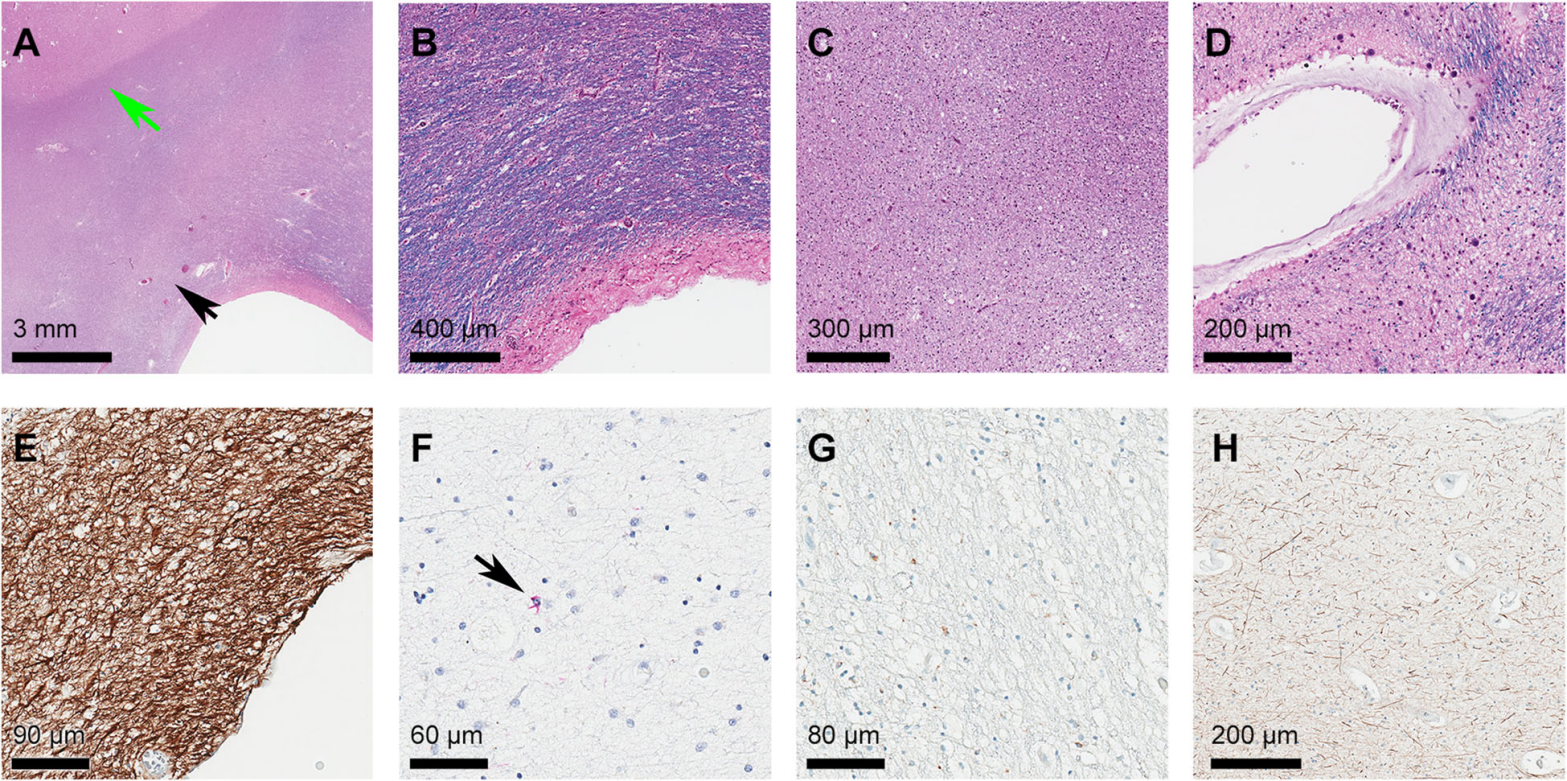
Selected examples of neuropathological findings in area of maximal white matter hyperintensity. **A** H&E/LFB staining of an FTLD-TDP case showing moderate (2) pallor of myelin staining within periventricular white matter (black arrow) in comparison to subcortical U fibers (green arrow); **B** H&E**/**LFB staining of an FTLD-tau (Pick’s) case showing pallor of periventricular myelin staining; **C** H&E/LFB staining of FTLD-tau (PSP) case showing severe myelin pallor; **D** H&E/LFB staining of an FTLD-tau (CBD) case showing collagenosis of large caliber periventricular veins; **E** GFAP immunolabeling of an FTLD-TDP case showing severe gliosis; **F** tau (AT8) immunohistochemistry of an FTLD-tau (CBD) case showing rare glial cytoplasmic tau immunopositive inclusions within the white matter region of interest (black arrow); **G** CD68 immunostaining of an FTLD-tau (Pick’s) case showing mild macrophage infiltration; **H** NF staining of an FTLD-tau (CBD) case showing severe axonal loss. *Abbreviations: AD* Alzheimer’s disease, *CAA* congophilic amyloid angiopathy, *CBD* corticobasal degeneration, *CD68* cluster of differentiation 68, *FTLD* frontotemporal lobar degeneration, *GFAP* glial fibrillary acid protein, *H&E/LFB* Hematoxylin Eosin with Luxol Fast Blue, *NF* neurofilament, *PSP* progressive supranuclear palsy

Cases of each pathologic neurodegenerative disease group with the highest burden of WMH on T2-weighted images were selected for further neuropathological assessment, and these results are shown in Fig. 3. All white matter regions of interest appeared normal macroscopically. The most frequent histologic correlate of WMH was the presence of moderate-severe gliosis on GFAP staining. Moderate-severe myelin pallor and axonal loss were also present in the majority of cases. Vascular pathology in the form of venous collagenosis was occasionally present, but no cases showed arteriolosclerosis, infarction, or perivascular hemosiderin (microbleeds). Macrophage infiltration was present in the majority of cases, but only in sparse amounts. All cases lacked TDP43 and tau (AT8) immunopositive inclusions, except for 1 case of FTLD-Tau (CBD) having very rare tau immunopositive glial inclusions. A summary of the pathological findings is provided in the Additional Table 2 (Additional file 1).

## Discussion

**Fig. 4:**
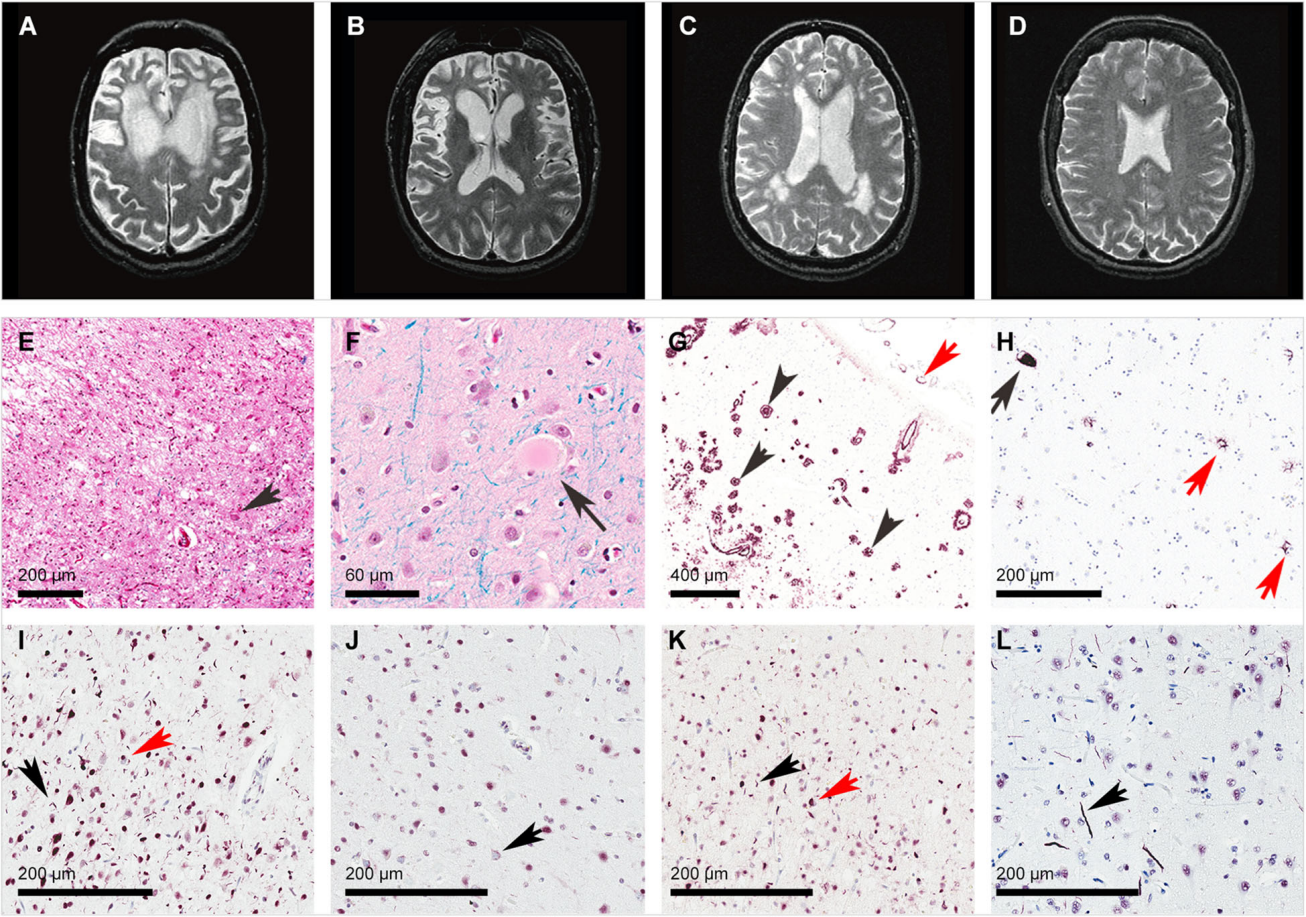
Selected examples of T2-weighted axial images and neurodegenerative neuropathological findings of participants. **A** T2-MRI of FTLD-TDP case with severe burden of WMH and prominent atrophy in medial and dorsolateral prefrontal cortex bilaterally; **B** T2-MRI of FTLD-tau (Pick's) case with prominent atrophy in medial and dorsolateral prefrontal cortex bilaterally with little WMH; **C** T2-MRI of AD case with WMH in posterior regions; and **D** T2-MRI of a healthy control. **E** H&E staining of FTLD-tau (Pick’s) case showing severe neuronal loss and gliosis in the frontal cortex with a ballooned neuron (black arrow); **F** H&E staining of FTLD-tau (CBD) case showing a ballooned neuron (black arrow) in frontal cortex; **G** beta-amyloid immunostaining of AD case showing frequent neuritic amyloid plaques (black arrows) and amyloid angiopathy (red arrow) in frontal cortex; and **H** tau (AT8) immunostaining of FTLD-tau (PSP) case showing a neurofibrillary tangle (black arrow) and astrocytic inclusions (red arrows). **I** FTLD-TDP Harmonized type A with short dystrophic neurites (black arrow) and compact neuronal cytoplasmic inclusions (red arrow) preferentially located in superficial cortical layers. **J** FTLD-TDP Harmonized type B with diffuse granular neuronal cytoplasmic inclusions (black arrow). **K** Mixed FTLD-TDP Harmonized A+B type with superficial dystrophic neurites (black arrow) and compact neuronal cytoplasmic inclusions (red arrow). **L** FTLD-TDP Harmonized type C with long thick dystrophic neurites (black arrow). Abbreviations: AD Alzheimer’s disease, CBD corticobasal degeneration, FTLD frontotemporal lobar degeneration, PSP progressive supranuclear palsy

We systematically analyzed and compared the volume and distribution of WMH seen on antemortem T2-weighted MRI in a cohort of neuropathologically proven cases of AD and FTLD and investigated the brain-behavioral associations between WMH and neuropsychiatric manifestations. We also reviewed the histologic findings within WMH in cases from each pathologic neurodegenerative disease group presenting the highest burden on brain imaging. We found a differential burden and varying distribution of WMH on T2-weighted MRI between these neuropathologies with cases of FTLD-TDP having a notably higher burden of WMH, particularly in frontotemporal regions, and AD cases having a higher burden of WMH in parieto-occipital regions (Fig. 4). In AD, concomitant presence of CAA was associated with a higher burden of WMH in the bilateral parietal regions. Moreover, we found that WMH burden correlated negatively with cortical volume in FTLD but not in AD, suggesting potentially different underlying neurobiological mechanisms. After controlling for several potential confounders, most importantly gray matter atrophy and multiple comparisons, we demonstrated that increased volume of WMH in distinct regions, notably in frontal white matter, was associated with greater neuropsychiatric manifestations as measured by the NPI. Lastly, neuropathological assessment of white matter regions with the highest burden of WMH on T2-weighted MRI revealed moderate to severe gliosis, myelin pallor, and axonal loss with minimal vascular pathology other than venous collagenosis.

### Mechanisms and distribution of WMH in AD and FTLD

The highest burden of WMH was found in FTLD-TDP cases. Extensive and widespread white matter involvement has been previously described in symptomatic *GRN* mutation carriers without significant vascular risk factors or other white matter diseases [29]. Although anomalies in white matter on diffusion tensor imaging have been described in chromosome 9 open reading frame 72 (*C9orf72)* mutation carriers [30], hyperintensities on T2-weighted or FLAIR imaging in white matter have not been reported for other known FTLD-causing genetic mutations. In the Genetic Frontotemporal Dementia Initiative (GENFI), only symptomatic *GRN* mutation carriers were found to have an increased global load of WMH when compared to normal controls, presymptomatic *GRN* mutation carriers, and both presymptomatic and symptomatic *C9orf72* and *MAPT* mutation carriers [31]. In the symptomatic *GRN* mutation subgroup, increased WMH burden was reported in the frontal and occipital lobes [31]. Although the precise mechanisms leading to white matter lesions in the context of progranulin deficiency are still not known, it has been hypothesized that progranulin’s functions in neuroinflammation and vasoprotection may play pivotal roles [32]. Interestingly in our cohort, FTLD-TDP cases were predominantly sporadic. While the underlying mechanisms leading to preferential involvement of the frontotemporal white matter and related neurocircuits still remain uncertain, the distribution of white matter lesions in our study appears to parallel areas of greater gray matter atrophy, suggesting common pathological factors, such as Wallerian degeneration.

In comparison to FTLD-TDP, FTLD-Tau (Pick’s) had a lower burden of WMH, suggesting that WMH load and distribution could have clinical utility in differentiating between these two proteinopathies that have overlapping clinical presentations. For FTLD-Tau (PSP) and FTLD-Tau (CBD), a few studies have described patterns of white matter atrophy as well as white matter anomalies on DTI [33, 34]. However, these studies have not systematically assessed nor reported the presence of WMH. Increased signal intensity changes on FLAIR images have been described in a small case series of patients with corticobasal syndrome with a higher burden noted more in frontal and parietal subcortical white matter, ipsilateral to the clinically affected hemisphere [35]. However, corticobasal syndrome is a pathologically heterogeneous group with cases due to CBD, PSP, TDP-43, Pick’s disease, and AD being described. Interestingly, in our cohort, FTLD-Tau (PSP) cases were found to have a significantly higher burden of WMH in parietal regions compared to healthy controls and to have a greater deep white matter burden than CBD cases, findings that have not been previously reported.

In contrast, white matter lesions in AD, such as signal changes and lacunar infarcts [9, 36], have been previously and extensively investigated and appear to be intricately interconnected with AD pathology [37]. WMH in AD have been attributed to periventricular small-vessel disease and neurodegenerative changes such as beta-amyloid deposition in arteries, arterioles, and veins and to contribute independently to brain atrophy and to onset of AD [8, 37]. WMH in AD have also been suggested to result from axonal loss secondary to cortical atrophy resulting from neuronal loss due to tau and beta-amyloid deposition [38]. Interestingly in our study, higher WMH load in the AD group was not associated with more severe cortical atrophy. Possible explanations for this finding include that WMH in AD may represent cerebral small vessel disease that preferentially affects deep branches, sparing superficial cortical branches early in the course of AD and that we adjusted for age in our model, controlling for the normal cortical atrophy seen with age. Moreover, the preferential distribution of WMH in periventricular regions in AD has been previously hypothesized to be linked to tissue properties such as a relatively lower normal perfusion of this region due to its location in watershed zones [8].

The histologic correlates within the WMH regions of interest, namely myelin pallor, axonal loss, and gliosis, were shared between all subjects, and there were no substantial differences in the white matter histology between pathologic disease groups. This may reflect the fact that only one case from each disease group underwent detailed pathologic analysis within the WMH region of interest. Therefore, additional work studying the histology within WMH in larger neuropathological case series is needed. The exact histologic footprint and pathogenesis of WMH has been somewhat elusive in the literature with some of the strongest associations to date being arteriolosclerosis and venous collagenosis associated with cerebrovascular risk factors. Since our cases were selected to minimize heterogeneity by exclusion of those with pathological comorbidity, this may be one reason why arteriolosclerosis and venous collagenosis were not seen. Questions left to be answered include the following: (1) In the absence of small vessel disease, are WMH simply epiphenomena of the underlying neurodegenerative process whatever that may be or are they associated with the specific underlying neuropathological substrate? (2) If they are epiphenomena only, why is the severity of WMH so variable across different neuropathologies?

### Association between WMH and manifestations of AD and FTLD

The significant positive correlations between WMH burden and neuropsychiatric manifestations on the NPI despite adjustment for gray matter volume suggest that WMH contribute independently to the clinical manifestations of AD and FTLD. WMH most likely contribute to neuropsychiatric manifestations across these neuropathologies by affecting the organization of networks. Only a few studies have specifically investigated brain-behavior relationships between WMH and neuropsychiatric manifestations in neurocognitive disorders, but no studies have focused on cases with autopsy confirmation of their neurodegenerative diagnosis. Similar to our results, a recent study in behavioral variant FTD and AD reported increased WMH in these disorders, partly independent of vascular pathology and cortical atrophy, with increased WMH being associated with cognitive deficits [39]. A study in participants with subcortical vascular cognitive impairment and AD reported an association between higher WMH volume in the frontal region with a higher level of apathy [40]. Similarly, a study in participants with probable AD reported increased WMH volumes in frontal regions for patients with apathy and increased WMH volumes in the right parietal region for those with depression [41]. In PSP, behavioral changes measured on the Frontal Behavioral Inventory (FBI) were found to correlate with atrophy in the orbitofrontal cortex and midbrain, but no significant correlations were identified with white matter disease [34]. However, comparable clinico-radiological correlations have been previously described in other neurological disorders, including multiple sclerosis. Findings of these studies highlight the contribution of WMH to neuropsychiatric manifestations across different clinical constructs.

### Strengths and limitations

A main strength of our study is the inclusion of neuropathologically proven cases of AD and FTLD, including both FTLD-tau and FTLD-TDP, which allowed us to study relationships between neuroanatomical locations of WMH and neuropsychiatric manifestations in pathologically confirmed neurodegenerative disease groups. We were also able to control for several factors associated with WMH in our models. By controlling for regional gray matter atrophy, we assessed the independent contribution of WMH to neuropsychiatric manifestations in these neuropathological entities. Nonetheless, there are limitations to acknowledge. First, genetic mutation carriers constituted only a small proportion of our cohort, and therefore, our results cannot be generalized to genetic cases of frontotemporal dementia or AD. Hence, we were unable to corroborate the potential differential effects of genetic mutations found in FTLD on neuroimaging findings that have been previously described [31]. As well, cases without co-existing neurodegenerative phenomena were selected for the present study, affecting generalization of findings to these highly prevalent mixed cases. While we included a remarkable number of pathology-proven cases, subgroup analyses were limited by the small sample size. Consequently, we could have missed other significant regional differences in WMH volumetrics between subgroups and subgroup-specific neural correlates of neuropsychiatric manifestations. Most statistically significant and robust differences and correlations identified in the present study pertain to the largest size subgroups, FTLD-TDP and AD. Conversely, while we adjusted our analyses for multiple comparisons using the false discovery rate, type I errors may not have been completely avoided. Finally, while we report several statistically significant correlations between WMH volumes, gray matter volumes, and neuropsychiatric manifestations in cross-sectional analyses, the temporal relationships between these variables remains to be further studied using longitudinal data before causation can be firmly ascribed.

## Conclusions

Our findings suggest that WMH seen on T2-weighted brain MRI are associated with neuropsychiatric manifestations in AD and FTLD and that WMH burden and regional distribution in neurodegenerative disorders differ according to the underlying neuropathological processes, where gliosis, myelin pallor, and axonal loss are prevalent pathological findings at autopsy. Future longitudinal studies need to be conducted to understand the temporal relationship between the occurrence of WMH and presentation with neuropsychiatric symptoms in FTLD and AD.

## Supplementary Information

**Additional file 1: Methods.** Manufacturer and dilution of employed immunohistochemical antibodies. Antibody information. **Table 1.** Individual clinical data. Demographic and clinical characteristics of participants according to neuropathological diagnosis. **Table 2.** Pathology review of selected area of white matter hyperintensity from each neuropathologic group. Semi-quantitative pathological findings. **Figure 1.** Example of image-guided neuropathology review of white matter regions with highest burden of WMH.

## Data Availability

The data that support the findings of this study are available from the corresponding author on reasonable request.

## Abbreviations

AD: Alzheimer’s disease
ANCOVA: Analysis of covariance
ANOVA: Analysis of variance
APP: Amyloid precursor protein
*C9orf72*: Chromosome 9 open reading frame 72
CAA: Congophilic amyloid angiopathy
CBD: Corticobasal degeneration
DRS: Dementia Rating Scale
DTI: Diffusion tensor imaging
FBI: Frontal Behavioral Inventory
FDR: False discovery rate
FTLD: Frontotemporal lobar degeneration
FTLD-Tau: Frontotemporal lobar degeneration due to tauopathy
FTLD-TDP: Frontotemporal lobar degeneration due to TDP-43 proteinopathy
GENFI: The Genetic Frontotemporal Initiative
*GRN*: Progranulin gene
HC: Healthy controls
LE: Lesion explorer
*MAPT*: Microtubule- associated protein tau gene
MMSE: Mini-Mental Status Examination
MRI: Magnetic resonance imaging
NPI: Neuropsychiatric Inventory
*PSEN1*: Presenilin-1 gene
*PSEN2*: Presenilin-2 gene
PSP: Progressive supranuclear palsy
SVD: Small-vessel disease
TDP-43: TAR DNA-binding protein 43
TIA: Transient ischemic attack
TIV: Total intracranial volume
WMH: White matter hyperintensity
