# Supplementary material for: White matter hyperintensities in autopsy-confirmed frontotemporal lobar degeneration and Alzheimer’s disease
Source: Alzheimers Res Ther. 2021 Jul 13;13:129. doi: 10.1186/s13195-021-00869-6 (PMC8278704; doi:10.1186/s13195-021-00869-6)
Supplement: Supplementary file 1 — Additional file 1: Methods. Manufacturer and dilution of employed immunohistochemical antibodies. Antibody information. Table 1. Individual clinical data. Demographic and clinical characteristics of participants according to neuropathological diagnosis. Table 2. Pathology review of selected area of white matter hyperintensity from each neuropathologic group. Semi-quantitative pathological findings. Figure 1. Example of image-guided neuropathology review of white matter regions with highest burden of WMH. [file 13195_2021_869_MOESM1_ESM.docx]

**Additional File 1**

| **Additional Methods: Manufacturer and dilution of employed immunohistochemical antibodies** | | |
| --- | --- | --- |
| **Immunohistochemical antibody** | **Manufacturer** | **Dilution** |
| Neurofilament | Ventana | Prediluted |
| GFAP | Ventana | Prediluted |
| CD68 | Ventana | Prediluted |
| Tau (AT8) | Thermofisher | 1/500 |
| p62 | BD lab | 1/200 |
| Alpha-synuclein | Invitrogen | 1/200 |
| TDP43 | Cederlane | 1/500 |

| **Additional Table 1: Individual clinical data** | | | | | | | | | | |
| --- | --- | --- | --- | --- | --- | --- | --- | --- | --- | --- |
| **Case** | **Neuropathological diagnosis** | **Sex** | **AAO (years)** | **Hand** | **Education (years)** | **Early symptoms** | **Disease duration (years)^a^** | **MMSE at baseline** | **DRS at baseline** | **Mutation carrier** |
| 1 | FTLD-TDP - B^b^ | F | 49.7 | R | 21 | Depression, disorganization | 3.8 | 26 | 114 | - |
| 2 | FTLD-TDP - C | F | 53.9 | R | 14 | Aphasia, episodic memory impairment | 11.8 | 25 | 111 | - |
| 3 | FTLD-TDP - C | F | 54.5 | L | 17 | Apathy, irritability, aphasia | 12.4 | 25 | 103 | - |
| 4 | FTLD-TDP - B | M | 55.0 | R | 13 | Episodic memory impairment, irritability | 3.5 | 30 | 126 | - |
| 5 | FTLD-TDP - A | M | 55.1 | R | 18 | Apathy, aphasia | 6.3 | 19 | 96 | *GRN+* |
| 6 | FTLD-TDP - A | M | 55.3 | R | 17 | Aphasia | 11.7 | 2 | 63 | - |
| 7 | FTLD-TDP - A | M | 56.8 | L | 12 | Apathy, irritability, aphasia | 7.3 | 23 | 116 | *C9orf72+* |
| 8 | FTLD-TDP^c^ | F | 58.8 | R | 16 | Aphasia, gaze palsy | 7.2 | 24 | 69 | *GRN+* |
| 9 | FTLD-TDP - C | F | 60.5 | R | 15 | Disinhibition, aphasia | 14.4 | 26 | 121 | - |
| 10 | FTLD-TDP - A | M | 61.8 | R | 18 | Apathy, irritability | 10.5 | 26 | 131 | *C9orf72+* |
| 11 | FTLD-TDP - A^b^ | M | 62.0 | L | 16 | Disinhibition, apathy, disorganization | 5.1 | 23 | 123 | *GRN+* |
| 12 | FTLD-TDP - A+B | F | 64.6 | R | 12 | Apathy, limb apraxia, depression | 9.3 | 20 | 92 | *GRN+* |
| 13 | FTLD-TDP - A | M | 65.2 | R | 16 | Limb apraxia and dystonia, anxiety | 6.7 |  |  | - |
| 14 | FTLD-TDP - C^b^ | F | 66.0 | A | 16 | Aphasia, apathy, disinhibition | 7.6 | 29 | 118 | - |
| 15 | FTLD-TDP - A | F | 66.0 | L | 18 | Apathy, stereotypical behaviors | 3.4 | 20 | 118 | - |
| 16 | FTLD-TDP^c^ | M | 67.0 | R | 19 | Apathy, episodic memory impairment | 15.3 | 29 | 144 | - |
| 17 | FTLD-TDP - A | M | 67.3 | R | 7 | Episodic memory impairment, aphasia | 17.1 | 23 | 100 | - |
| 18 | FTLD-TDP - A+B | M | 68.5 | R | 16 | Anxiety, apathy, aphasia | 5.9 | 22 | 97 | - |
| 19 | FTLD-TDP - A | F | 71.1 | R | 13 | Episodic memory impairment, aphasia | 6.2 | 20 | 83 | - |
| 20 | FTLD-TDP - A | F | 75.5 | R | 13 | Gait difficulties, limb apraxia | 5.7 | 12 |  | - |
| Blank cells indicate testing not performed.  AAO = age at onset; DRS = Dementia rating scale; Hand = handedness: R = right-handed; L = left-handed; A = ambidextrous; MMSE = mini-mental state examination.  ^a^ Disease duration represents the number of years from age at onset to death.  ^b^ Case selected for additional pathologic review.  ^c^ Harmonized subtype was unavailable. | | | | | | | | | | |

| ***Additional Table 1. continued*** | | | | | | | | | | | | |
| --- | --- | --- | --- | --- | --- | --- | --- | --- | --- | --- | --- | --- |
| **Case** | **Neuropathological diagnosis** | **Sex** | **AAO (years)** | **Hand** | **Education (years)** | **Early symptoms** | **Disease duration (years)^a^** | | **MMSE at baseline** | **DRS at baseline** | | **Mutation carrier** |
| 21 | FTLD-TDP - A | M | 79.2 | R | 12 | Apathy, disinhibition, hyperorality | 16.2 | | 26 | 125 | | - |
| 22 | FTLD-TDP^c^ | M | 80.2 | R | 11 | Episodic memory impairment | 13.5 | | 23 | 121 | | - |
| 23 | FTLD-tau (Pick's) | F | 45.9 | L | 13 | Aphasia | 19.9 | | 3 | 48 | | - |
| 24 | FTLD-tau (Pick's)^b^ | M | 55.4 | L | 17 | Apathy, disinhibition, irritability | 11.7 | | 29 | 134 | | - |
| 25 | FTLD-tau (Pick's) | M | 55.7 | R | 16 | Aphasia | 16 | | 1 | 57 | | - |
| 26 | FTLD-tau (Pick's) | M | 58.5 | R | 12 | Aphasia, aggressivity | 15.8 | | 19 | 113 | | - |
| 27 | FTLD-tau (Pick's) | F | 59.0 | R | 23 | Aphasia, euphoria, disinhibition | 3.9 | | 27 | 142 | | - |
| 28 | FTLD-tau (Pick's) | M | 60.4 | A | 20 | Aphasia, Anxiety | 21.1 | | 5 |  | | - |
| 29 | FTLD-tau (Pick's) | F | 67.8 | R | 15 | Episodic memory impairment | 10.9 | | 14 | 62 | | - |
| 30 | FTLD-tau (Pick's) | M | 71.8 | R | 15 | Delusions, apathy, depression | 9.3 | | 27 | 138 | | - |
| 31 | FTLD-tau (Pick's) | F | 75.1 | R | 15 | Episodic memory impairment, disinhibition | 6.5 | | 20 | 111 | | - |
| 32 | FTLD-tau (Pick's) | M | 80.6 | R | 8 | Depression, disinhibition, delusions | 5.1 | | 24 | 118 | | - |
| 33 | FTLD-tau (CBD) | M | 52.0 | R | 12 | Apathy, disinhibition, aphasia | 8.5 | | 26 | 124 | | - |
| 34 | FTLD-tau (CBD) | F | 56.9 | L | 16 | Disinhibition, aphasia, memory impairment | 13.2 | | 22 | 86 | | - |
| 35 | FTLD-tau (CBD) | F | 61.5 | R | 9 | Aphasia, limb apraxia, balance difficulties | | 3.4 | 15 | | 70 | - |
| 36 | FTLD-tau (CBD) | M | 62.5 | R | 14 | Aphasia, episodic memory impairment | 3.2 | | 16 | 118 | | - |
| 37 | FTLD-tau (CBD) | F | 62.5 | R | 13 | Limb apraxia and dystonia | 6.5 | | 26 | 69 | | - |
| 38 | FTLD-tau (CBD) | F | 62.6 | R | 12 | Aphasia, limb apraxia | 9.8 | | 16 | 83 | | - |
| 39 | FTLD-tau (CBD)^b^ | F | 66.7 | R | 12 | Aphasia, apathy, irritability | 7.5 | | 13 |  | | - |
| 40 | FTLD-tau (CBD) | F | 67.0 | R | 10 | Aphasia, emotional lability | 9.4 | | 26 | 118 | | - |
| Blank cells indicate testing not performed.  AAO = age at onset; DRS = Dementia rating scale; Hand = handedness: R = right-handed; L = left-handed; A = ambidextrous; MMSE = mini-mental state examination.  ^a^Disease duration represents the number of years from age at onset to death.  ^b^ Case selected for additional pathologic review.  ^c^ Harmonized subtype was unavailable. | | | | | | | | | | | | |

| ***Additional Table 1. continued*** | | | | | | | | | | |
| --- | --- | --- | --- | --- | --- | --- | --- | --- | --- | --- |
| **Case** | **Neuropathological diagnosis** | **Sex** | **AAO (years)** | **Hand** | **Education (years)** | **Early symptoms** | **Disease duration (years)^a^** | **MMSE at baseline** | **DRS at baseline** | **Mutation carrier** |
| 41 | FTLD-tau (CBD) | M | 68.0 | R | 20 | Aphasia, disinhibition | 8.8 | 28 | 137 | - |
| 42 | FTLD-tau (CBD) | F | 68.6 | R | 17 | Apathy, aphasia, eating habit changes | 2.7 | 24 | 99 | - |
| 43 | FTLD-tau (CBD) | F | 74.1 | R | 13 | Apathy, limb apraxia and dystonia, dysarthria | 7.2 | 29 | 130 | - |
| 44 | FTLD-tau (PSP) | F | 55.2 | R | 12 | Parkinsonism, apathy, limb apraxia | 10.3 | 28 | 109 | - |
| 45 | FTLD-tau (PSP) | M | 62.4 | R | 19 | Parkinsonism, falls, apathy | 7.9 | 28 | 116 | - |
| 46 | FTLD-tau (PSP) | M | 63.0 | R | 13 | Parkinsonism, apathy, irritability, apraxia | 10.5 | 20 | 88 | - |
| 47 | FTLD-tau (PSP)^b^ | F | 64.5 | R | 12 | Parkinsonism, delusions, anxiety, apathy | 5.8 | 27 | 109 | - |
| 48 | FTLD-tau (PSP) | M | 64.5 | R | 16 | Parkinsonism, apathy, limb apraxia | 7.7 | 27 | 121 | - |
| 49 | FTLD-tau (PSP) | F | 66.6 | R | 13 | Episodic memory impairment, agitation | 11.4 | 18 | 108 | - |
| 50 | FTLD-tau (PSP) | M | 67.1 | R | 14 | Parkinsonism, aphasia | 8.3 | 26 | 126 | - |
| 51 | FTLD-tau (PSP) | F | 68.5 | R | 14 | Aphasia, paranoia, limb apraxia | 8.7 | 8 |  | - |
| 52 | FTLD-tau (PSP) | M | 71.1 | A | 11 | Spastic dysarthria, falls, parkinsonism | 9.9 | 21 | 99 | - |
| 53 | FTLD-tau (PSP) | M | 71.2 | R | 20 | Disinhibition, parkinsonism, aphasia | 10.1 | 29 | 135 | - |
| 54 | FTLD-tau (PSP) | F | 71.3 | R | 16 | Episodic memory impairment, parkinsonism | 3.7 | 17 | 75 | - |
| 55 | FTLD-tau (PSP) | F | 72.1 | L | 19 | Parkinsonism, agitation | 8.6 | 30 | 131 | - |
| 56 | FTLD-tau (PSP) | M | 74.2 | R | 18 | Parkinsonism, anxiety | 7.5 | 30 | 131 | - |
| 57 | FTLD-tau (PSP) | M | 75.2 | R | 12 | Parkinsonism | 2.9 | 26 | 126 | - |
| 58 | FTLD-tau (PSP) | M | 77.7 | R | 20 | Parkinsonism, apathy | 7.4 | 27 | 142 | - |
| 59 | AD^b^ | F | 50.4 | R | 16 | Episodic memory impairment, apathy | 8.3 | 20 | 117 | - |
| 60 | AD | M | 52.2 | R | 20 | Episodic memory impairment | 5.2 | 13 | 59 | - |
| Blank cells indicate testing not performed.  AAO = age at onset; DRS = Dementia rating scale; Hand = handedness: R = right-handed; L = left-handed; A = ambidextrous; MMSE = mini-mental state examination.  ^a^Disease duration represents the number of years from age at onset to death.  ^b^ Case selected for additional pathologic review.  ^c^ Harmonized subtype was unavailable. | | | | | | | | | | |

| ***Additional Table 1. continued*** | | | | | | | | | | |
| --- | --- | --- | --- | --- | --- | --- | --- | --- | --- | --- |
| **Case** | **Neuropathological diagnosis** | **Sex** | **AAO (years)** | **Hand** | **Education (years)** | **Early symptoms** | **Disease duration (years)^a^** | **MMSE at baseline** | **DRS at baseline** | **Mutation carrier** |
| 61 | AD | M | 53.5 | R | 10 | Episodic memory impairment | 11.9 | 18 | 125 | - |
| 62 | AD | F | 59.2 | R | 12 | Episodic memory impairment, mental rigidity | 11.8 | 27 | 111 | - |
| 63 | AD | M | 61.0 | R | 15 | Episodic memory impairment | 8 | 16 | 92 | - |
| 64 | AD | F | 63.7 | R | 26 | Episodic memory impairment, apathy | 16.4 | 29 | 139 | - |
| 65 | AD | M | 64.9 | R | 28 | Episodic memory impairment | 8.4 | 28 | 133 | - |
| 66 | AD | F | 65.0 | R | 12 | Episodic memory impairment, apathy | 10.2 | 21 | 117 | - |
| 67 | AD | M | 65.2 | R | 16 | Episodic memory impairment, apathy | 5.1 | 26 | 116 | - |
| 68 | AD | M | 65.8 | R | 10 | Episodic memory impairment | 8.8 | 10 | 87 | - |
| 69 | AD^b^ | M | 68.5 | R | 10 | Episodic memory impairment, aphasia | 11.8 | 12 |  | - |
| 70 | AD | M | 71.1 | R | 12 | Episodic memory impairment | 4.4 | 26 | 133 | - |
| 71 | AD | M | 72.3 | R | 16 | Episodic memory impairment, apathy | 12.8 | 20 | 100 | - |
| 72 | AD | M | 80.9 | R | 19 | Episodic memory impairment, hallucinations | 3.9 | 24 | 108 | - |
| 73 | AD | F | 89.9 | R | 18 | Episodic memory impairment | 8.3 | 25 | 115 | - |
| Blank cells indicate testing not performed.  AAO = age at onset; DRS = Dementia rating scale; Hand = handedness: R = right-handed; L = left-handed; A = ambidextrous; MMSE = mini-mental state examination.  ^a^Disease duration represents the number of years from age at onset to death.  ^b^ Case selected for additional pathologic review.  ^c^ Harmonized subtype was unavailable. | | | | | | | | | | |

| **Additional Table 2: Pathology review of white matter hyperintensity regions of interest from each neuropathologic group** | | | | | | | | | | | | |
| --- | --- | --- | --- | --- | --- | --- | --- | --- | --- | --- | --- | --- |
| **Stain** | | **H&E/LFB** | | | | | | **NF** | **GFAP** | **CD68** | **TDP43** | **Tau** |
| **Neuropathological diagnosis** | **Periventricular white matter brain region assessed** | **Myelin pallor** | **Arteriolo-sclerosis** | **Infarcts/**  **microinfarcts** | **Hemosiderin** | **Large caliber venous collagenosis** | **Small caliber venous collagenosis** | Axonal loss | Gliosis | Macrophage/ microglia | Inclusions | Inclusions |
| FTLD-TDP - A | L frontal | 2 | No | No | No | Yes | No | 1 | 1 | 1 | 0 | 0 |
| FTLD-TDP - B^a^ | R frontal | - | - | - | - | - | - | - | - | - | - | - |
| FTLD-TDP - C | R frontal | 2 | No | No | No | No | No | 3 | 3 | 0 | 0 | 0 |
| FTLD-tau (Pick's) | L frontal | 0 | No | No | No | No | No | 1 | 3 | 1 | 0 | 0 |
| FTLD-tau (CBD) | R frontal | 3 | No | No | No | Yes | Yes | 3 | 3 | 1 | 0 | 1 |
| FTLD-tau (PSP) | L parietal | 2 | No | No | No | No | No | 2 | 3 | 0 | 0 | 0 |
| AD without CAA | R frontal | 2 | No | No | No | Yes | Yes | 2 | 2 | 0 | 0 | 0 |
| AD with CAA | L frontal | 1 | No | No | No | No | No | 1 | 3 | 1 | 0 | 0 |

Semi-quantitative scale: 0 = None; 1 = Mild; 2 = Moderate; 3 = Severe.

CD68 = Cluster of Differentiation 68; GFAP = Glial fibrillary acid protein; H&E/LFB = Hematoxylin Eosin with Luxol Fast Blue; NF = Neurofilament; TDP43 = TAR DNA binding protein 43

^a^Excluded as no WMH region of interest was identified in the remaining cadaveric formalin fixed brain tissue or in the tissue section.

**Additional Figure 1**

**
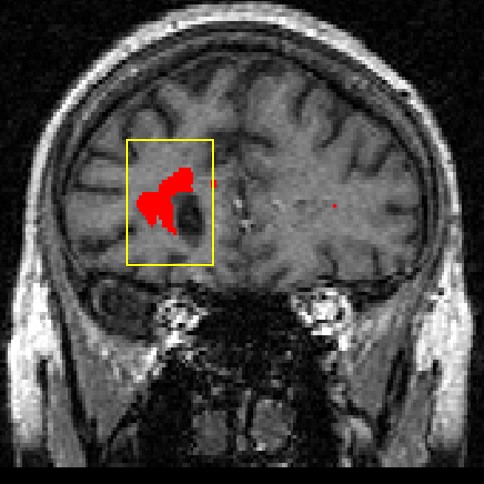
**

**Example of image-guided neuropathology review of white matter regions with highest burden of WMH.**

Annotated coronal MRI image guide to identify brain region with highest burden of white matter hyperintensity for further additional pathologic review.
